# Activation of Coagulation by Lenalidomide-Based Regimens for the Treatment of Multiple Myeloma

**DOI:** 10.1371/journal.pone.0064369

**Published:** 2013-05-16

**Authors:** Yu Isozumi, Reina Arai, Kazumi Fujimoto, Takatoshi Koyama

**Affiliations:** Graduate School of Health Care Sciences, Tokyo Medical and Dental University, Tokyo, Japan; INRS, Canada

## Abstract

We investigated the procoagulant effects of lenalidomide (Len)-based regimens *in vitro* focusing on tissue factor (TF) and phosphatidylserine (PS). We examined the effects of a pharmacological concentration of Len with or without the corticosteroid dexamethasone (Dex) and the proteasome inhibitor bortezomib (Bor) using the human vascular endothelial cell line EAhy926 and the monocytic cell lines THP-1 and U937. Cell-surface procoagulant activity (PCA) was induced by Dex-containing regimens in all lines. Expression of TF antigen on the cell surface and of TF mRNA was markedly increased by Dex-containing regimens. PS exposure was increased modestly by a Len-based regimen. PS exposure was increased modestly in EAhy926 cells, and markedly increased in THP-1 and U937 cells by Bor-containing treatment. An anti-TF monoclonal antibody almost completely blocked the induced PCA. When Len is given in combination with Dex, PCA may be induced on endothelial cells and monocytes through TF expression and PS exposure.

## Introduction

Multiple myeloma (MM) is characterized by the proliferation of clonal plasma cells in the bone marrow. Several combinations of chemotherapy and/or autologous peripheral blood stem cell transplantation have been undertaken in MM patients. Achieving a good prognosis has been difficult, so the development of new therapeutic agents has been undertaken.

Recently, the new antineoplastic drugs thalidomide (Thal), lenalidomide (Len) and bortezomib (Bor) have been used to improve the response rates in the treatment of MM. Combination with corticosteroids enhances the tumoricidal effects [1], [2], [3], [4].

Len is a structural analog of Thal. Thal and Len were initially used for the treatment of relapsed/refractory MM, but both immunomodulatory drugs are now being included in frontline regimens. Len has more potent antimyeloma properties than Thal and lacks many of its unfavorable features (including neurotoxicity). Len: has tumoricidal effects; interferes with the plasma cell–microenvironment interaction with subsequent downregulation of the crucial cytokines required for the growth of plasma cells; has immune stimulatory properties [2].

Dexamethasone (Dex) is a corticosteroid, and is similar to a natural hormone produced by the adrenal glands. Dex acts as an anti-inflammatory immunosuppressant and as a lymphotoxic agent. Combination therapies of Thal or Len with Dex and chemotherapeutic agents have led to high response rates [3], but deep-vein thrombosis (DVT) has been noted as one of the most serious side effects with these regimens [5], [6]. DVT is caused by blood congestion, vascular damage and accentuation of blood coagulation. It leads to circulatory disturbance or edema, and further complicates pulmonary thromboembolism (PTE), which can be lethal. The cause of DVT under treatment using Len with corticosteroids or chemotherapeutic agents is not clear.

Proteasome inhibition has also been explored extensively as a therapeutic strategy in MM, and proteasome inhibitors now form a cornerstone of anti-MM therapy. Bor was the first-in-class proteasome inhibitor introduced into the clinic [4]. The most common hematologic toxicity associated with Bor is transient thrombocytopenia. Bor does not seem to accelerate the thrombotic tendency associated with immunomodulatory drugs such as Len and Thal [4].

Tissue factor (TF) has a key role in coagulation factors because it catalyzes the conversion of the inactive protease factor X/XI into the active protease factor Xa/IXa. TF is constitutively expressed in several extravascular cells, but is not normally expressed in cells that come into contact with blood. Monocytes and vascular endothelial cells can express TF in response to pathological stimuli. Phosphatidylserine (PS) localizes at the inner leaflet of the bilayer, but becomes exposed if the membrane structure collapses by apoptosis and is thought to be connected with TF de-encryption [7]. PS activates the coagulation cascade [8]. Therefore, increased exposure to PS may result in upregulated procoagulant activity (PCA).

Recently, we reported that Thal-based regimens with Dex and doxorubicin induced PCA in human vascular endothelial cells and monocytic cells but did not induce PCA and expression of TF in myeloma cells [9]. Neoplastic plasma cells do not express TF [10]. So we investigated further the procoagulant effects of Len-based regimens with Dex and Bor in human venous endothelial cells and monocytic cells, focusing on TF and PS.

## Methods

### Reagents

(±)-Len (Selleck Chemicals, Houston, TX, USA) was dissolved in dimethyl sulfoxide (DMSO) and then added to media at 7.4×10^−7^ M. Dex was dissolved in ethanol (EtOH) and then added to media at 5.0×10^−7^ M. Bor was dissolved in ddH_2_O and then added to media at 1.6×10^−8^ M. As an untreated control, the same amount of DMSO, EtOH and ddH_2_O were added to the culture medium. Concentrations of Len, Bor, and high-dose Dex that are used in the clinic were added to media simultaneously and incubated for 24 h. We referred to the interview forms of Len (Revlimid*®*), Bor (Velcade*®*), and Dex (LenaDex*®*) and associated reports for the concentrations [11], [12], [13], and selected the median plasma concentrations.

All reagents were of reagent grade and purchased from Wako Pure Chemicals (Osaka, Japan) unless otherwise indicated.

### Cell culture

As a model of endothelial cells within blood vessels, we used the human umbilical vein endothelial cell line EAhy926 [14]. EAhy926 were cultured in Dulbecco's modified Eagle's medium (DMEM) with high glucose media supplemented with 10% fetal bovine serum (FBS; Biological Industries, Kibbutz Beit Haemek, Israel) and 50 U/mL penicillin-streptomycin. EAhy926 was kindly provided by Dr. Edgell (University of North Carolina, Chapel Hill, NC, USA). As a model of monocytes, the monocytic leukemia cell lines THP-1 and U937 were cultured in RPMI1640 media supplemented with 10% FBS and 50 U/mL penicillin-streptomycin. THP-1 and U937 were from Health Science Research Resources Bank (Osaka, Japan). We could not obtain sufficient numbers of vascular endothelial cells and peripheral blood monocytes for the assays, so we used these cell lines in the study.

### PCA assay

An aliquot of cells (2×10^6^) was suspended in phosphate-buffered saline (PBS), and added to 0.1 mL of pooled normal human plasma. After incubation at 37°C for 2 min, 0.1 mL of 25 mM calcium chloride was added and the plasma recalcification time measured using a CA-100 Semiautomatic Coagulator (Sysmex, Kobe, Japan). PCA was measured quantitatively by reference to a standard curve constructed with human placental TF. The amount of TF activity that yielded a 50-s recalcification time was defined as 1 U/mL, as described previously [15].

### Cell-surface TF antigens (flow cytometric analyses)

All cell lines were incubated with Len, Dex and Bor for 24 h. Cell suspensions in PBS were first incubated with a monoclonal anti-TF antibody (American Diagnostica, Greenwich, CT, USA) and then with a fluorescein isothiocyanate (FITC)-labeled anti-mouse IgG antibody (Beckman Coulter, Fullerton, CA, USA) for 60 min on ice. After washing with PBS, they were run on a Flow Cytometer (FACScan, Becton Dickinson, San Diego, CA, USA) using acquisition and analysis software (CELLQuest, Becton Dickinson).

### Quantitative reverse transcription-polymerase chain reaction (RT-PCR) and densitometric analyses

All cell lines were incubated with Len, Dex and Bor for 4 h. Total cellular RNA was isolated using a High Pure RNA Isolation Kit (Roche Diagnostics, Mannheim, Germany). RT-PCR assay was carried out using a Titan One Tube RT-PCR Kit (Roche Diagnostics) according to manufacturer instructions. cDNA derived from each cell was amplified by 28 PCR cycles. The relative signal intensity of bands was determined and standardized using Scion Image Software, as described previously [15].

### Cell-surface PS exposure (flow cytometric analyses)

Suspensions of each cell line in 1×Annexin V Binding Buffer (Beckman Coulter) were incubated with FITC-labeled annexin V (Beckman Coulter) for 15 min on ice and with propidium iodide (PI) (Beckman Coulter). Immediately after the antibody reaction, aliquots were run on a Flow Cytometer (FACScan) using acquisition and analysis software (CELLQuest).

### Cell-surface PCA after incubation with anti-TF antibody

To investigate the effect of cell-surface TF on induced PCA by a Len-based regimen, samples were preincubated with 1.96 µg/mL 6B4 (American Diagnostica), monoclonal mouse anti-human TF IgG, or the same amount of irrelevant IgG, in PBS for 60 min on ice. After washing with PBS, cell-surface PCA was assessed as described above.

### Statistical analyses

Data are the means ± S.D. Statistical analyses were carried out using the non-parametric Mann–Whitney U test for paired data using GraphPad Prism 5 (GraphPad Software, San Diego, CA, USA). Significant differences are indicated (O or *) with (*P*<0.05) compared with control and Len alone, respectively, whereas (OO or **) indicates a significant difference with (*P*<0.01), and (OOO or ***) with (*P*<0.001) compared with control and Len alone.

## Results

### Effects of Len, Dex, and Bor on cell surface PCA

**Figure 1:**
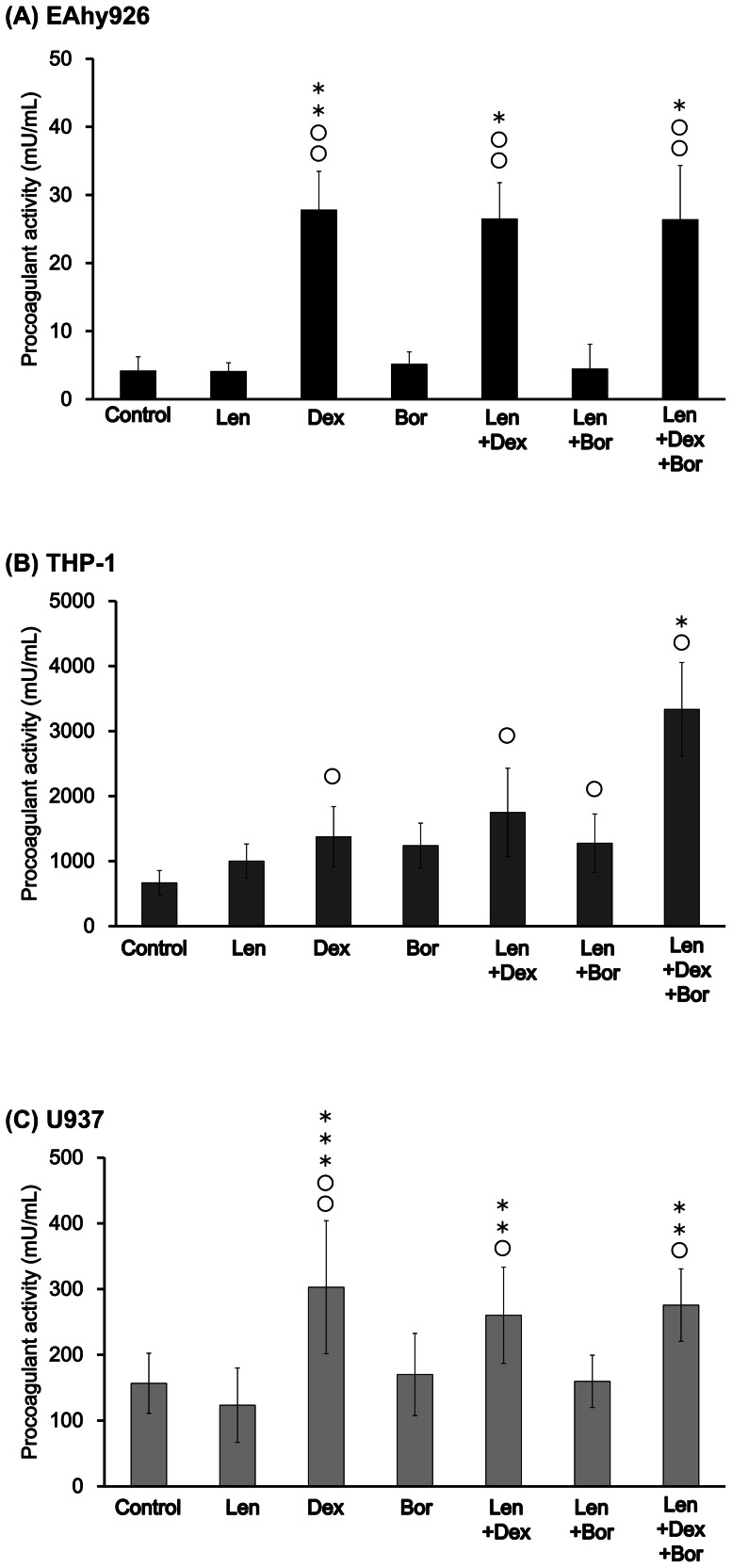
Effects of Len, Dex, and Bor on cell-surface PCA. Each sample (A: EAhy926, B: THP-1, C: U937) was incubated with Len (7.4×10^−7^ M), Dex (5.0×10^−7^ M), Bor (1.6×10^−8^ M), Len/Dex, Len/Bor, and Len/Dex/Bor, respectively, at 37°C for 24 h. PCA was measured by normal-plasma based recalcification time. Data are the mean ± SD (n = 6). Significant differences are indicated by (O or *) with (*P*<0.05) compared with control and Len alone, respectively, whereas (OO or **) indicates a significant difference with (*P*<0.01), and (OOO or ***) with (*P*<0.001) compared with control and Len alone.

We examined whether Len, Dex and Bor could increase cell-surface PCA in EAhy926, THP-1 and U937 cells (Fig. 1).

In EAhy926 cells stimulated with a Dex-containing regimen, PCA was upregulated significantly compared with control. This upregulation was induced by Dex because stimulation with Len or Bor alone did not induce PCA (Fig. 1A).

In THP-1 cells stimulated with Dex alone, PCA was induced significantly compared with control. Co-incubation of Len with Dex and/or Bor significantly upregulated PCA compared with control or Len alone (Fig. 1B).

In U937 cells, cell-surface PCA was increased by Dex alone. Co-incubation of Len with Dex and Dex/Bor further upregulated PCA compared with control and Len alone. This upregulation was induced by Dex because stimulation with Len or Bor alone decreased or did not change PCA, respectively (Fig. 1C)

### Effects of Len, Dex and Bor on the expression of cell-surface TF antigen

**Figure 2:**
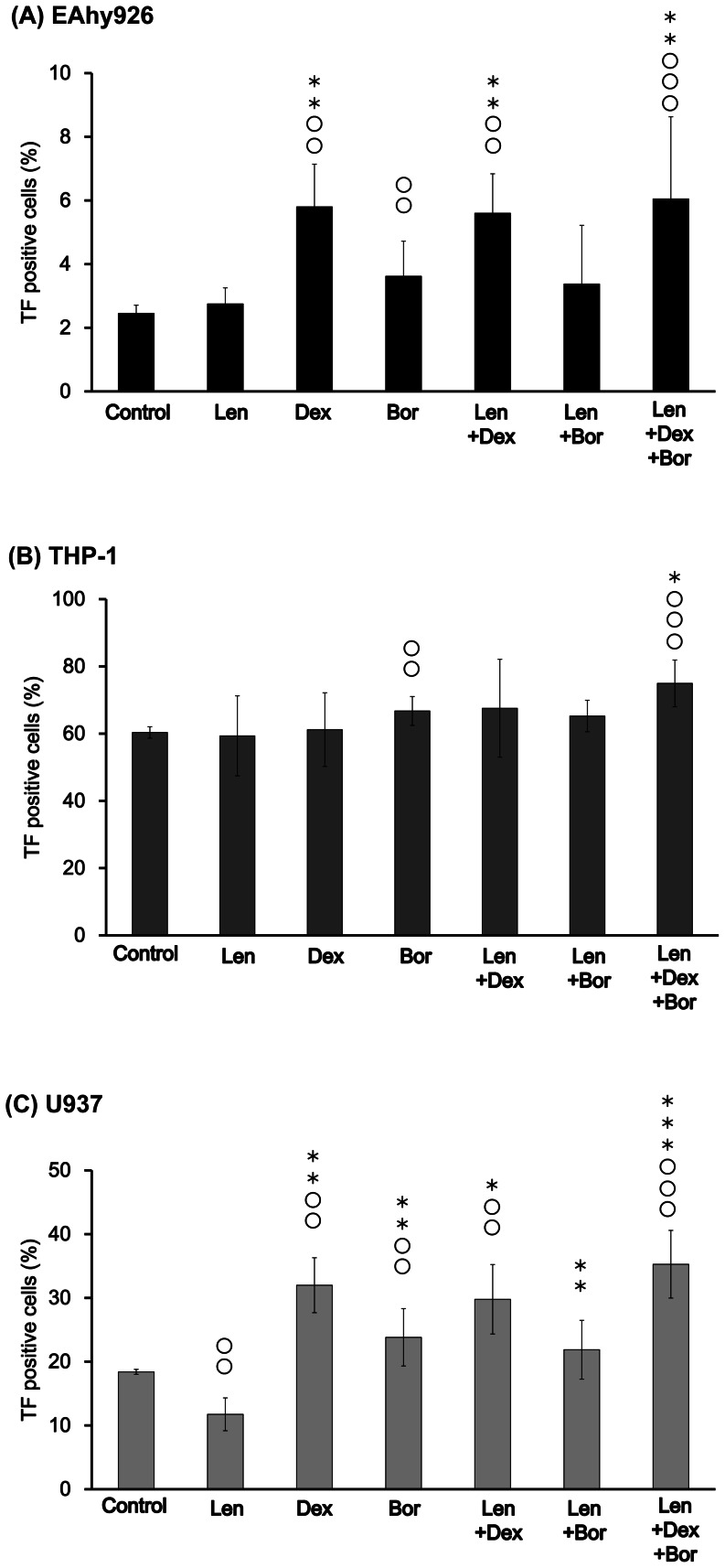
Effects of Len, Dex, and Bor on cell-surface expression of TF antigen. Each sample (A: EAhy926, B: THP-1, C: U937) was incubated with Len (7.4×10^−7^ M), Dex (5.0×10^−7^ M), Bor (1.6×10^−8^ M), Len/Dex, Len/Bor, and Len/Dex/Bor, respectively, at 37°C for 24 h. Cell-surface expression of TF antigen was analyzed by flow cytometry. Data are the mean ± SD (n = 6). Significant differences are indicated by (O or *) with (*P*<0.05) compared with control and Len alone, respectively, whereas (OO or **) indicates a significant difference with (*P*<0.01), and (OOO or ***) with (*P*<0.001) compared with control and Len alone.

We examined if Len, Dex, and Bor could increase the expression of TF antigen on the cell surface in EAhy926, THP-1 and U937 cells by flow cytometry (Fig. 2).

The level of TF antigen on EAhy926 and U937 cells was increased by Dex, Bor, and co-incubation of Len with Dex and Dex/Bor (Fig 2A and C). In U937 cells, TF antigen exposure was decreased by Len alone.

In THP-1 cells, cell-surface TF antigen was not affected by Len and Dex except for co-incubation with Len, Dex and Bor (Fig. 2B).

### Effects of Len, Dex and Bor on TF mRNA

TF mRNA levels were quantified by RT-PCR assay to reveal the transcriptional stimulus for producing TF in EAhy926, THP-1 and U937 cells.

**Figure 3:**
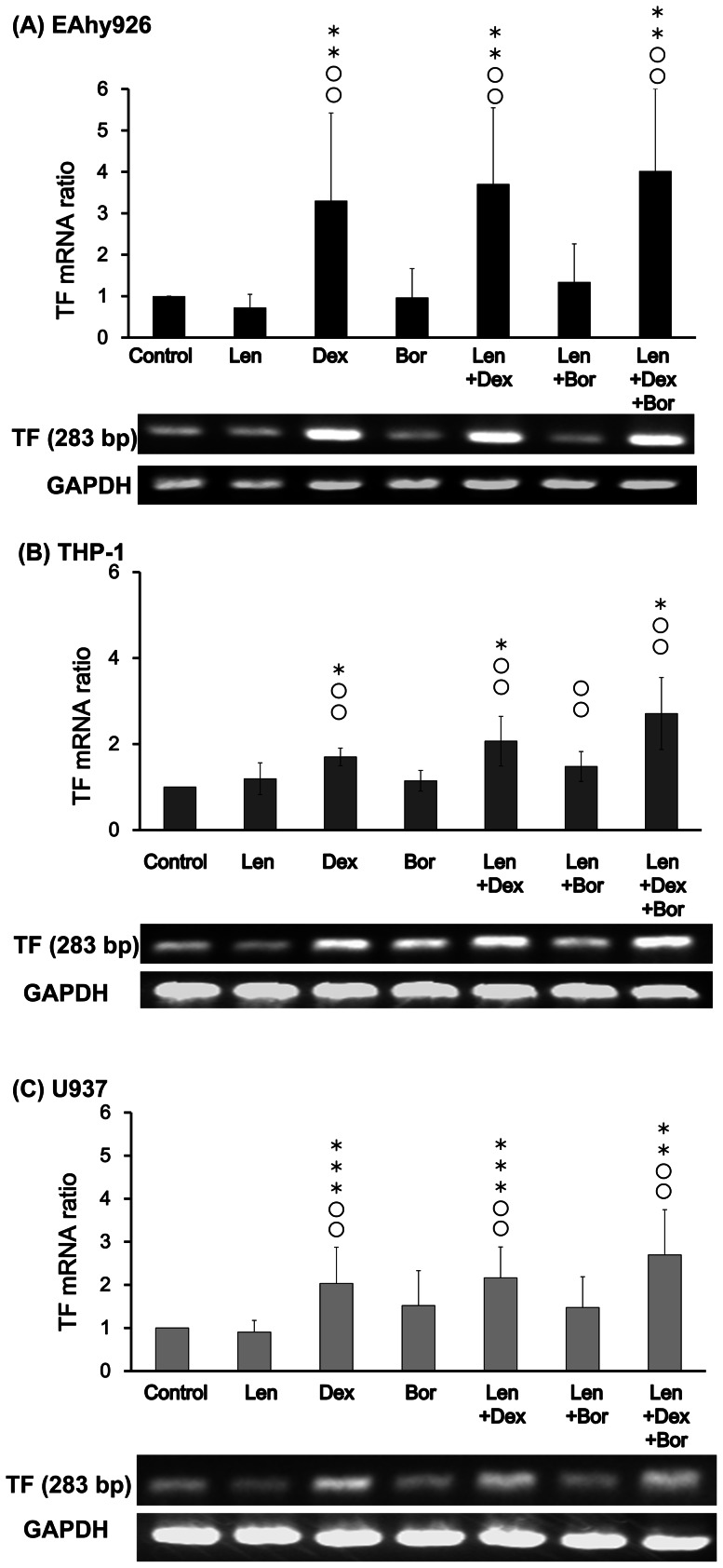
Effects of Len, Dex, and Bor on TF mRNA. Each sample (A: EAhy926, B: THP-1, C: U937) was incubated with Len (7.4×10^−7^ M), Dex (5.0×10^−7^ M), Bor (1.6×10^−8^ M), Len/Dex, Len/Bor, and Len/Dex/Bor, respectively, at 37°C for 4 h. Total RNA was extracted and stocked until RT-PCR. cDNA derived from all cell lines was amplified by 28 cycles. Glyceraldehyde 3-phosphate dehydrogenase (GAPDH) mRNA was used as an mRNA quantity control. Relative signal intensity was determined and data are the mean ± SD (n = 6).

In EAhy926 cells stimulated with Dex-containing regimens, TF mRNA was increased significantly compared with control and Len alone (Fig. 3A).

In THP-1 cells, TF mRNA was increased by stimulation with Dex and co-incubation of Len with Dex and/or Bor compared with control and Len alone (Fig. 3B).

In U937 cells, stimulation with Dex alone led to a significant increase in TF mRNA compared with control. Co-incubation of Len with Dex or Dex/Bor led to further increases compared with Len alone and control (Fig. 3C).

These results suggested that Dex induced upregulation of TF mRNA because Len or Bor alone did not significantly change levels of TF mRNA.

### Effects of Len, Dex and Bor on PS exposure on the cell surface

Annexin V combines with PS with high compatibility in the phospholipid bilayer (which includes PS). Therefore, an increase in the combination of annexin V suggests that alterations in the plasma membrane have been induced and coagulation might be promoted [16], [17].

**Figure 4:**
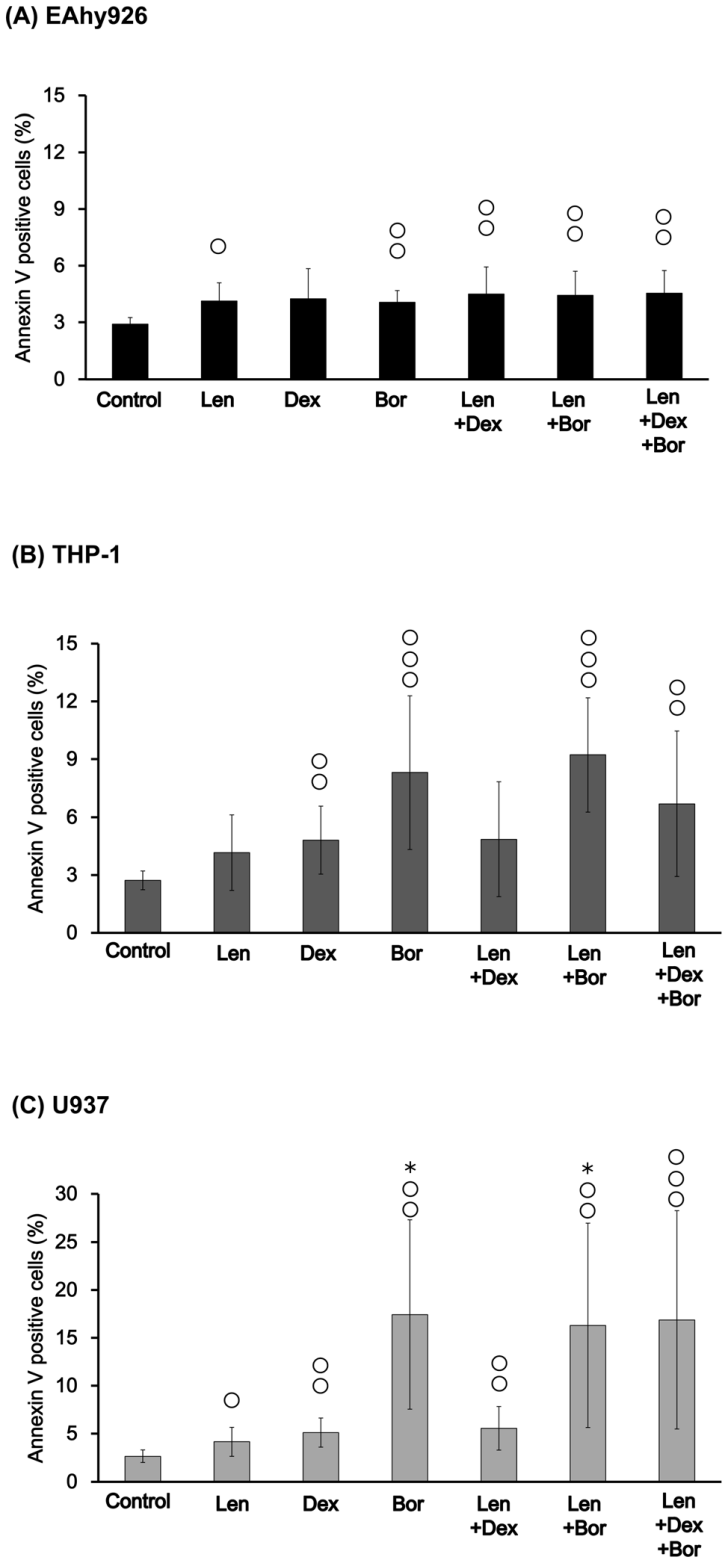
Effects of Len, Dex, and Bor on PS exposure. Each sample (A: EAhy926, B: THP-1, C: U937) was incubated with Len (7.4×10^−7^ M), Dex (5.0×10^−7^ M), Bor (1.6×10^−8^ M), Len/Dex, Len/Bor, and Len/Dex/Bor, respectively, at 37°C for 24 h. Cell-surface expression of PS was analyzed by flow cytometry. PS was detected by FITC-labeled annexin V.

In EAhy926 cells stimulated with Bor alone, PS exposure was increased compared with control, and co-incubation of Len with Dex and/or Bor also increased such exposure (Fig. 4A).

Exposure to PS on the surface of THP-1 and U937 cells was increased significantly by Bor alone, co-incubation of Len with Bor and Dex/Bor compared with control, and by Len alone. Cell-surface exposure to PS was increased modestly by Len, Dex and Len/Dex compared with control (Figs 4B and C).

### Effects of Len, Dex and Bor on cell-surface PCA blocked by anti-TF antibody

**Figure 5:**
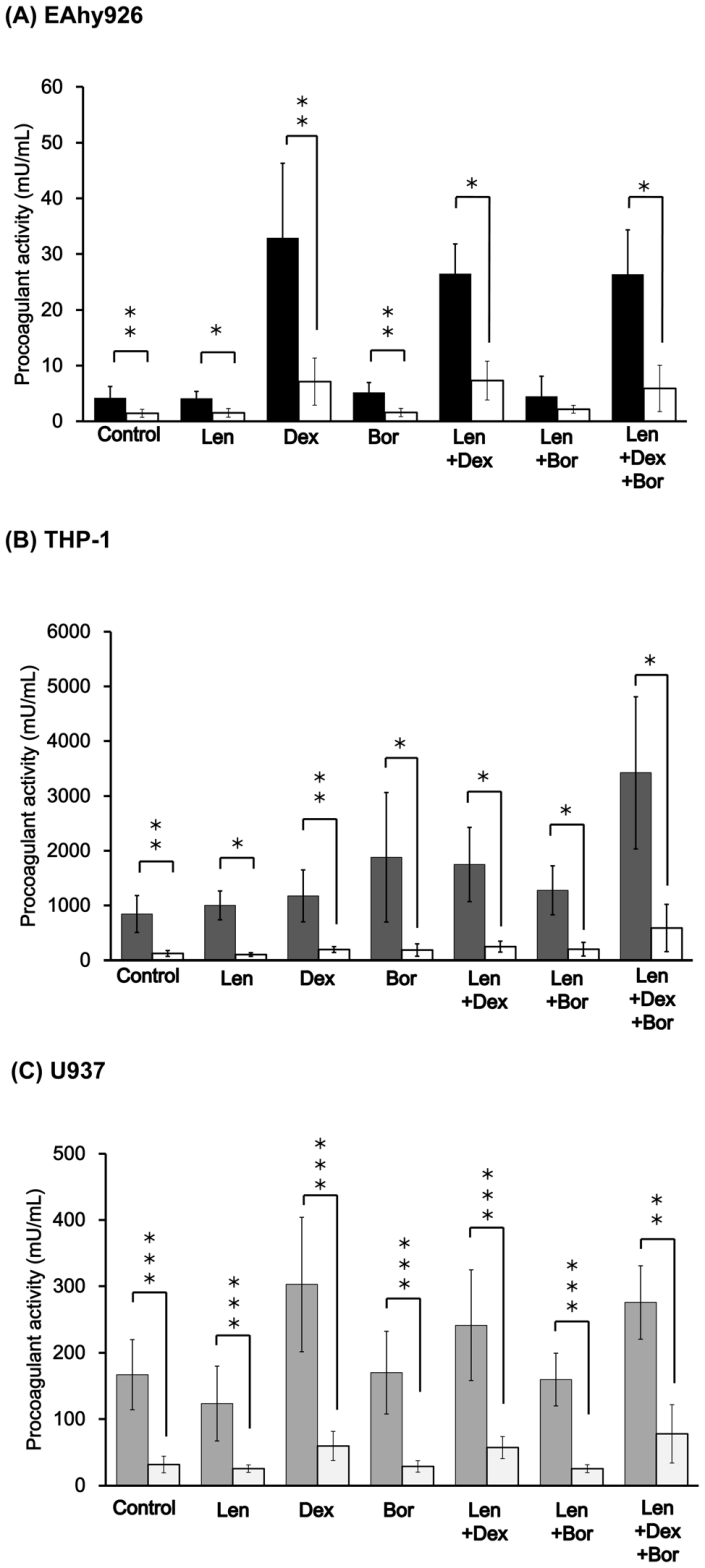
Effects of an anti-TF antibody on cell-surface PCA. To investigate the effect of cell-surface TF on induced PCA by a Len-based regimen, samples were preincubated with 1.96 µg/mL 6B4, monoclonal mouse anti-human TF IgG, or the same amount of irrelevant IgG, in PBS for 60 min on ice. After washing with PBS, cell-surface PCA was assessed as in Fig. 1. Data are the mean ± SD (n = 5). Significant differences are indicated (*) with (*P*<0.05), whereas (**) indicates a significant difference with (*P*<0.01), and (***) with (*P*<0.001).

In all cell lines, upregulated cell-surface PCA was blocked significantly by anti-TF antibody 6B4. This blocking was obvious in samples stimulated with Dex-containing regimens. These results suggested that the increase in PCA by Dex-containing regimens was induced by upregulation of the expression and activity of TF on the cell surface (Fig. 5).

## Discussion

We showed here that Dex alone and co-incubation of Len with Dex and Dex/Bor significantly increased PCA on the cell surface of vascular endothelial and monocytic cells.

The absence of an increased risk of DVT in MM patients treated with Thal or Len as single agents suggests that these drugs are not directly thrombogenic. Hence, combination effects with other agents or other disease-specific factors have appeared to significantly contribute to the risk. In fact, the combination of Len with Dex was shown to increase the prevalence of DVT in newly diagnosed MM patients by 8–75% and in relapsed/refractory patients by 8–16% [5]. Recently, Valsami et al. reported that Thal or Len used as single agents increased the endothelial expression of TF induced by the inflammatory cytokine tumor necrosis factor-α (TNFα) [18]. Aue et al. reported that Len induced the release of many proinflammatory cytokines in patients with chronic lymphocytic leukemia (CLL), including TNFα, and that inflammation, TNFα and endothelial dysfunction might link Len to DVT in patients with CLL [19]. However, those inflammatory side effects were not observed in patients with MM or lymphoma.

To investigate why PCA was increased by Len-based combination regimens in the vasculature of patients with MM, we focused on the expression of TF and PS on the surface of vascular endothelial and monocytic cells.

In EAhy926 cells, expression of cell-surface TF antigen was increased remarkably by Dex-containing regimens compared with control and Len alone. TF mRNA was upregulated in a similar fashion. Induced cell-surface PCA was blocked significantly by an anti-TF antibody. Therefore, Dex treatment may induce the expression of TF antigen and increase PCA.

In THP-1 cells, cell-surface TF antigen was not significantly affected by treatment with Len, Dex and Bor except for co-incubation of Len/Dex/Bor. However, the level of TF mRNA was increased by Dex-containing regimens, and the modestly induced PCA was blocked by an anti-TF antibody. This discrepancy might be because malignant THP-1 cells constitutively express a huge amount of cell-surface TF antigen even before stimulation with reagents.

In U937 cells, expression of TF antigen on the cell surface and TF mRNA were increased remarkably by Dex-containing regimens compared with control and Len. Bor treatment increased the level of TF antigen, but TF mRNA levels did not change significantly.

PS may activate the coagulation cascade. PS exposure on apoptotic cells may appear to promote the activation of TF and factor VIIa complex [7].

In EAhy926 cells, PS exposure was increased modestly by Bor treatment. In THP-1 and U937 cells, an appreciable increase to PS exposure was demonstrated, which may suggest the antitumor activity of Bor. These results may also be associated with an increase in PCA.

The mitogen-activated protein kinase signaling pathway and the downstream regulated immediate-early gene *Egr1* seem to be the molecular targets of activated glucocorticoid receptors [20]. Recently, we suggested that Dex accelerated the Egr-1 pathway to induce TF expression in EAhy926 cells [9]. The lower toxicity associated with lower-dose Dex (principally DVT and infections) resulted in a superior one-year overall survival in the Len/lower-dose Dex (Dex 40 mg, p.o., weekly) arm than in the Len/conventional high-dose Dex (Dex 40 mg, p.o., daily, days 1–4, 9–12, 17–20 every 28 days) arm [3].

Len is known to have an antiangiogenic activity. In blood vessels, Len may disrupt repair of the injured endothelium by other anti-tumor agents, strengthen the effects of the other drugs, and extend the induced PCA activity.

Bor modestly induced PS exposure on endothelial cells and monocytic cells *in vitro*, but Bor did not inhibit the PCA induced by Dex. When a combination of Len/Dex/Bor is applied in the clinic, Bor does not seem to accelerate the thrombotic tendency associated with Len/Dex [21]. Hiroi et al. reported that proteasome inhibitors such as Bor enhanced the expression of an anticoagulant glycoprotein thrombomodulin in endothelial cells [22]. However, we did not observe an enhancement of thrombomodulin expression in the cells that we used (data not shown).

Len is widely used in combination with other agents not only for MM, but also for other hematological malignancies. We need to understand the duality of the actions of these drugs on malignant clones and the surrounding microenvironment. We suggest that prophylactic anticoagulant strategies should be considered in Len-based regimens monitoring procoagulant markers such as D-dimers [6], [23]. A small amount of aspirin with antiplatelet activity [24] may not be sufficient for prophylaxis in high-dose Dex-containing Len-based regimens.
